# Insight into the Sustainable Integration of Bio- and Petroleum Refineries for the Production of Fuels and Chemicals

**DOI:** 10.3390/polym12051091

**Published:** 2020-05-11

**Authors:** Wegik Dwi Prasetyo, Zulfan Adi Putra, Muhammad Roil Bilad, Teuku Meurah Indra Mahlia, Yusuf Wibisono, Nik Abdul Hadi Nordin, Mohd Dzul Hakim Wirzal

**Affiliations:** 1 Chemical Engineering Department, Universiti Teknologi PETRONAS, Seri Iskandar 32610, Perak, Malaysia; wegik_16003931@utp.edu.my (W.D.P.); nahadi.sapiaa@utp.edu.my (N.A.H.N.); mdzulhakim.wirzal@utp.edu.my (M.D.H.W.); 2 Department of Chemical Engineering, Universitas Pertamina, Jl.Teuku Nyak Arief, Simprug, Kebayoran Lama, Jakarta 12220, Indonesia; 3 PETRONAS Group Technical Solutions, Project Delivery and Technology, PETRONAS, Kuala Lumpur 50050, Malaysia; zulfan.adiputra@petronas.com; 4 School of Information, Systems and Modelling, Faculty of Engineering and Information Technology, University of Technology Sydney, Sydney, NSW 2007, Australia; TMIndra.Mahlia@uts.edu.au; 5 Bioprocess Engineering, Faculty of Agricultural Technology, Brawijaya University, Malang 65141, Indonesia; y_wibisono@ub.ac.id

**Keywords:** biomass, biorefinery, process optimization, mathematical programming deterministic, stochastics, single-objective optimization, multi-objective optimization

## Abstract

A petroleum refinery heavily depends on crude oil as its main feedstock to produce liquid fuels and chemicals. In the long term, this unyielding dependency is threatened by the depletion of the crude oil reserve. However, in the short term, its price highly fluctuates due to various factors, such as regional and global security instability causing additional complexity on refinery production planning. The petroleum refining industries are also drawing criticism and pressure due to their direct and indirect impacts on the environment. The exhaust gas emission of automobiles apart from the industrial and power plant emission has been viewed as the cause of global warming. In this sense, there is a need for a feasible, sustainable, and environmentally friendly generation process of fuels and chemicals. The attention turns to the utilization of biomass as a potential feedstock to produce substitutes for petroleum-derived fuels and building blocks for biochemicals. Biomass is abundant and currently is still low in utilization. The biorefinery, a facility to convert biomass into biofuels and biochemicals, is still lacking in competitiveness to a petroleum refinery. An attractive solution that addresses both is by the integration of bio- and petroleum refineries. In this context, the right decision making in the process selection and technologies can lower the investment and operational costs and assure optimum yield. Process optimization based on mathematical programming has been extensively used to conduct techno-economic and sustainability analysis for bio-, petroleum, and the integration of both refineries. This paper provides insights into the context of crude oil and biomass as potential refinery feedstocks. The current optimization status of either bio- or petroleum refineries and their integration is reviewed with the focus on the methods to solve the multi-objective optimization problems. Internal and external uncertain parameters are important aspects in process optimization. The nature of these uncertain parameters and their representation methods in process optimization are also discussed.

## 1. Introduction

To achieve sustainable economic growth, industries require safe and sustainable feedstock [1]. While the economy of energy has various resources (water, wind, solar heating and light, geothermal heat, biomass, nuclear fission, and fusion), the material bioeconomy depends highly on biomass. Research and development are necessary to achieve following objectives: (i) increase the scientific understanding of biomass resources and valorize the applications of those resources, (ii) improve sustainable system to develop, harvest, and process biomass resources, (iii) improve efficiency and performance in conversion and distribution processes and technologies for a host of bio-based products development, and (iv) create the regulatory and market environment necessary for increased development and use of bio-based products [2]. 

The paradigm-shift from the oil-based to the renewable resources-based economy must be supported by the research and developments. Research ensures continuous development of technology and the valorization of innovation. Nonetheless, the renewable resources-based economy faces many challenges. The biggest challenge is the conversion technology, which is required to be technically feasible, economically viable, and environmentally safe. Processes and technologies have been developed to utilize biomass as the feedstock for production of fuels and chemicals [3]. The facility that processes biomass into fuels and chemicals is known as a biorefinery. The biorefinery that processes the first-generation biomass (sugar and lipid) of edible crop plant into fuels have been well established. However, the conflicting interest of fuel vs. food and fuel vs. feed has triggered the rising price of the first-generation biomass commodity. Agricultural crop residue, wood, and manure belong to the second-generation biomass class, while microalgae is classified as the third-generation biomass [4]. The second- and third-generation biomasses are potential sources of biorefinery feedstock. In the designing of a new process for the utilization of biomass, a process simulation is a powerful tool. Well-constructed process simulation provides valuable data of the operating condition, material balance, and energy requirement [5,6,7,8,9,10]. The data then can be further utilized in process optimization. The optimization task is not limited to finding the optimum condition related to a chemical process but also to find an optimum supply chain network. An efficient supply chain network is also required in a highly competitive economy due to the scarcity of resources. The logistics costs in the agricultural sector include material transportation and distribution, inventory, and information process costs [11]. The high logistics cost in the agricultural sector is proportional to the volume and quantity. The discontinuous availability of biomass due to spatial fragmentation and seasonal variability also leads to an increase in the logistics cost. Furthermore, a supply chain network is characterized by a high degree of uncertainty, where the value of some parameters cannot be controlled by the decision maker [12]. Uncertainty is defined as the difference between the amount of required information and its availability to execute the task. It is related to the decision-making process under incomplete information. It can be classified into randomness, epistemic, and deep uncertainty. Randomness arises from the random nature of the events and concerns with the membership or non-membership of an element in a set. 

The decision-making process in the optimization of a biorefinery should consider several operational and strategic uncertainties [13]. Many studies in biorefinery supply chain optimization involve a deterministic approach that ignores the uncertainty. The deterministic approach is commonly used in plant planning and scheduling, typically resulting in an overly conservative or over-aggressive decision. An over-conservative decision leads to unnecessary profit loss while an aggressive decision may result in severe constraints violation [14]. Failure to account for the uncertainty ultimately causes faulty decisions and inadequate strategies, leading to failure to capitalize on the opportunities. The non-deterministic approaches that accounted for the uncertainties have attracted great interest in the field of process optimization. The commonly used non-deterministic approaches are stochastic programming, robust optimization, and fuzzy programming. Stochastics programming is a common approach to deal with randomness, and thus is suitable to address the issues related to bio- and petroleum refineries. The epistemic uncertainty is caused by the lack of data or information on the parameters. Possibilistic programming is widely used to study epistemic uncertainty [15]. The solutions attained are based on the estimate of the actual condition and thus carry some risk. Deep uncertainty can be dealt with robust convex programming approaches. If there is a combination of randomness, epistemic, and deep uncertainty in the model, fuzzy-interval possibilistic programming or full-infinite fuzzy stochastic programming can also be employed. While the stochastics-based process optimization is powerful, it is exhaustive when the number of uncertainty and its samples grow larger. The sources of uncertainties frequently studied in the nondeterministic biorefinery supply chains include biomass supply, product demand, product price, and technology development [13]. The studies on the optimization of biorefinery under uncertainty are dominantly multi-objective with the economic potential and the environmental impact as the objectives. The maximization of the net present value (NPV), the maximization of profit, and the minimization of the total cost are commonly applied for the economic analysis, while the environmental impact objective is often quantified through Eco-indicator 99 or global warming potential. 

This work discloses the context of feedstocks in bio- and petroleum refineries. The issue associated with crude oil supplies and price instability is highlighted to show the importance of feedstock diversification by considering the ones from biomass. Later, the important characteristics of biomass that allow it as a potential feedstock for refinery are also elaborated, including the potential end-product chemicals or intermediate that can bridge biomass processing facilities with the refineries. A section is dedicated to comparing the chemical composition of different biomass sources and their characterization. Such comparison is important because it influences the choice of process and technology to convert biomass. The locality-availability aspect of biomass is also considered by presenting the most potential biomass source in Malaysia. A comprehensive overview of the current progress of mathematical programming-based process optimization is also presented. It highlights the prominent issues that arise in the developed model and approached strategies, including the main uncertainty factors that affect the optimization. 

## 2. The Depleting Reserves and Price Instability of Global Crude Oil 

**Figure 1:**
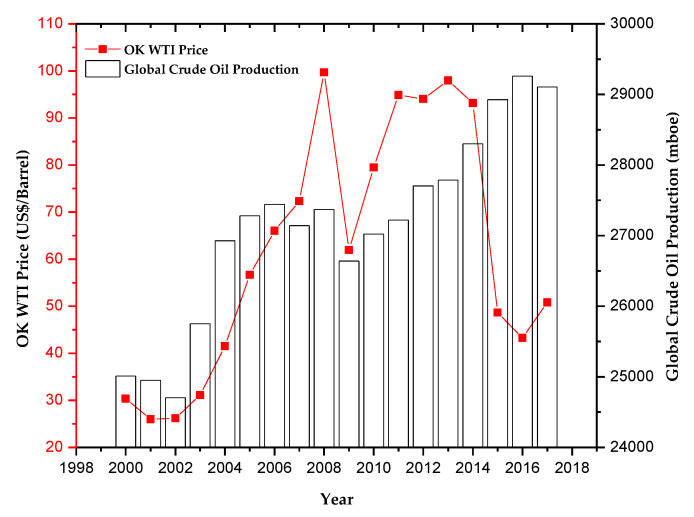
Crude oil price fluctuation (red line) and the annual global crude oil production rate (bar chart). OK WTI: West Texas Intermediate Crude Oil Traded in Oklahoma, adapted from [18,27].

Fuels and petrochemicals are substantive materials that enable our modern-way of life. They are the derivative products of crude oil and natural gas converted through a refinery and chemical processing facilities. Recently, some have witnessed the volatility of crude oil prices due to several factors, namely the depleting crude oil reserves, global and regional geopolitical instability, the ever-increasing demand, and many others [16] (Figure 1). The nations to suffer the most are the ones who depend on importing crude oil and natural gases. For such countries, it is a necessity to diversify the feedstocks of the fuels and petrochemical refineries. According to the data from the Organisation for Economic Co-operation and Development (OECD), the global proven crude oil reserves are 1482.77 bboe (billion barrels of oil equivalent) [17] and the global production rate of crude oil is about 3.97 million of ktoe (kilotonne of oil equivalent) or equals to 29,100 million of boe (barrel of oil equivalent) [18]. It follows that the global crude oil reserves will last roughly within 51 years by assuming that there is no fluctuation in the annual crude oil production rate and no new reserve is discovered. 

The biorefinery concept emerges in response to these challenges. It is defined as a facility with an integrated, efficient, and flexible conversion processes of biomass feedstocks, through a combination of physical, chemical, biochemical, and thermochemical processes, into multiple products such as fuels, chemicals, materials, and/or heat and power [19,20,21,22]. Such an approach is not new. Many industrial materials used at the beginning of the 20th century, such as dyes, solvents, synthetic fibers, and fuel, were made from trees and crops [23,24,25]. However, many of the bio-based chemical products had been displaced by petroleum derivatives by the late 1960s. Shifting the dependence of society away from petroleum to renewable biomass resources, including microalgae, is viewed as an important contributor to the development of a sustainable industrial society [22]. 

Few countries have issued directives to gradually decrease the dependence on petroleum. The U.S. Department of Energy has set goals to replace 30% of the liquid petroleum transportation fuel with biofuels and to replace 25% of industrial organic chemicals with biomass-derived chemicals by 2025 [2]. The revised Renewable Energy Directive of the European Union (EU) requires the countries within the EU to fulfill at least 27% of the EU’s total energy needs with renewable by 2030. It further specifies that all countries within the EU must also ensure that at least 10% of their transport fuels come from renewable sources by 2020 [26]. 

The decline of petroleum and natural gas reserves as the current main sources of fuels and chemicals has led to the ever-growing commitment to the establishment of a bioresources-based economy.

## 3. Biomass as a Renewable Biorefinery Feedstock

Biorefineries require sustainable and renewable resources. An ideal renewable resource is one that can be replenished over a relatively short timescale or is abundant [28]. Biomass fits this definition. It is a renewable feedstock that can be utilized to produce fuels and chemicals [29]. Biomass is any organic matter that is available on a renewable or recurring basis (excluding old-growth timber), including dedicated energy crops and trees, agricultural food and feed crop residues, animal wastes, and other waste materials [30]. In contrast with the limited nature and composition of petrol resources, bioresource and biomass are a collection of gathered compounds of very different natures, namely cellulose, hemicellulose, oils, lignin, and so on [16].

### 3.1. Plant Biomass Composition

Understanding the biomass composition is very important as a basis for bio- and petroleum refinery integration. The intermediate products from biomass as feedstock to a petroleum refinery should have a composition close to the current refinery feedstock. Plants capture solar energy as fixed carbon, converting CO_2_ and water to sugars. The produced sugar is stored in three different types of polymer: cellulose, hemicellulose, and starch [31]. Biomass is typically composed of 65–85 wt % sugar-based polymers (principally cellulose and hemicellulose), with another 10–25% corresponding to lignin. Other biomass minor components include triglycerides, sterols, alkaloids, resins, terpenes, terpenoids, and waxes (often collectively referred to as lipids), as well as inorganic minerals. In the case of seeds and certain algae strains, significant amounts of oil can present, corresponding mainly to triglycerides. This is exemplified by soybeans (ca. 20 wt % oil), rapeseed (ca. 40 wt % oil), and oil-palm fruit (ca. 50 wt % oil), which together account for the majority of the feedstock currently used in biodiesel production. Together, cellulose, hemicellulose, and lignin constitute lignocellulose, which is the fibrous materials that form the cell walls of plants and trees. 

**Figure 2:**
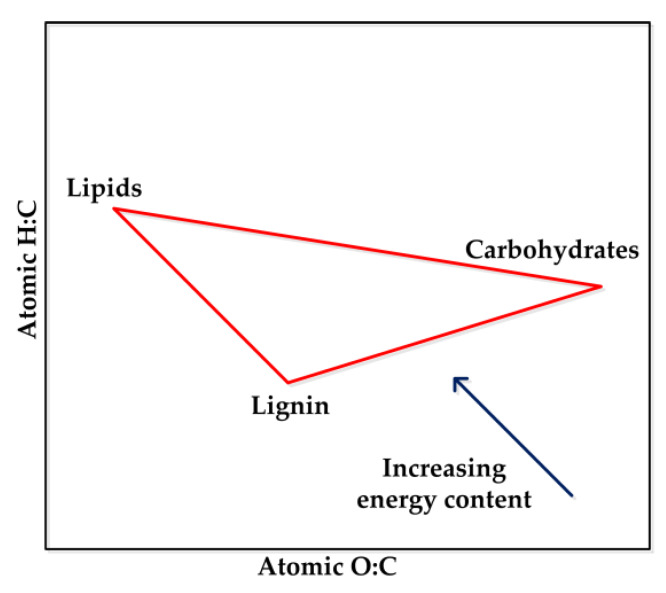
Elemental Composition of Biomass (van Krevelen plot) [32] – Reproduced by permission of The Royal Society of Chemistry.

The elemental composition of sugars, lignin, and lipids in biomass can be described by the van Krevelen plot (Figure 2). The energy content of biomass constituents depends on the oxygen content and the hydrogen to carbon (H:C) ratio. A biomass constituent with lower oxygen content and higher H:C ratio will have higher energy content. Hence, energy content per unit of mass follows the order of lipids > lignin > sugars. 

Sugar polymers such as cellulose and starch can be readily broken down by hydrolysis for the conversion to ethanol or other chemicals. Lignin is harder to be broken down due to its relative chemical inertness. Current bioethanol production is mostly based on the fermentation of sugars that are readily obtained from the starch in corn grain, in addition to the sugar in sugarcane and sugar beets. Even though lipids and sugars are the ideal starting material for biofuels production, the future large-scale production of biofuels will have to be based on the utilization of lignocellulose as the principal feedstock due to its relative abundance. A further advantage of lignocellulose utilization is the avoidance of the conundrum of “foods vs. fuels”.

### 3.2. Biomass Characterization

**Figure 3:**
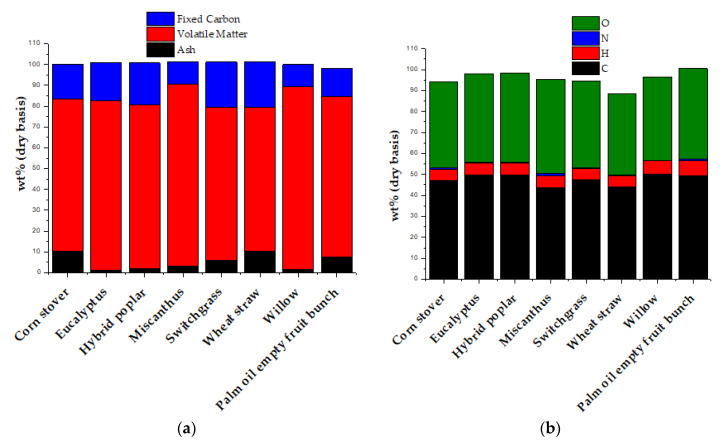
Characterization of Biomass: (**a**) Proximate Analysis and (**b**) Ultimate Analysis, adapted from [32,33].

Biomass can originate from a multitude of sources with high variability. Proper characterization is thus important before the selection processing technology. The most important biomass characterization comprises of proximate and ultimate composition (typically shown in Figure 3), heating value, and the process of biomass production, collection, storing, transporting, and processing (crop yields, economic, equipment availability, grinding performance, etc.).

Proximate analysis is based on the change of the product under controlled heating; it is comprised of moisture content, volatile matter, fixed carbon, and ash. On the other hand, the ultimate analysis quantifies the elements that constitute the biomass, typically carbon, hydrogen, oxygen, nitrogen, sulfur, and chlorine. This analysis also quantifies the moisture content and ash. Figure 3 shows the ultimate and proximate analysis of several biomass feedstocks.

Biomass can also be grouped by the energy contained within the chemical bonds in the biomass. The higher heating value (HHV) and lower heating value (LHV) are the parameters used to quantify the energy content. The term gross calorific value is used interchangeably as HHV, while the net calorific value is interchangeably used in describing LHV. HHV is the amount of heat released from the combustion including the latent heat of vaporization of water from the sample. LHV is the measured heat released excluding the contribution of the latent heat of vaporization. 

**Figure 4:**
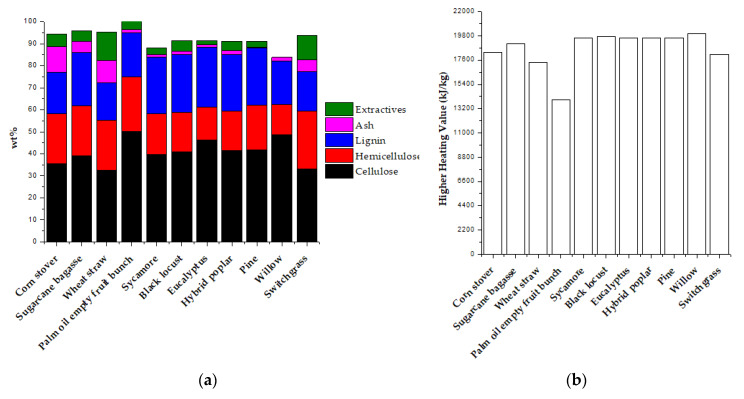
(**a**) Compositions of Biomass Expressed as a Percentage of Bone-Dry Material, and the (**b**) Higher Heating Value, adapted from [33,34].

The biomass that has a higher content of cellulose and hemicellulose relative to the lignin is preferred for biochemical conversion to ethanol. Meanwhile, the higher content of lignin and extractives in the biomass is desirable for the thermochemical conversion process. The heating value of lignin and extractives is higher than the cellulose and hemicellulose. Figure 4 summarizes the composition of various feedstocks and their HHV. The lignin-based biomass can also be used to produce intermediate chemicals (i.e., pyrolysis oil) that can be used as feedstock for the petroleum refinery.

**Figure 5:**
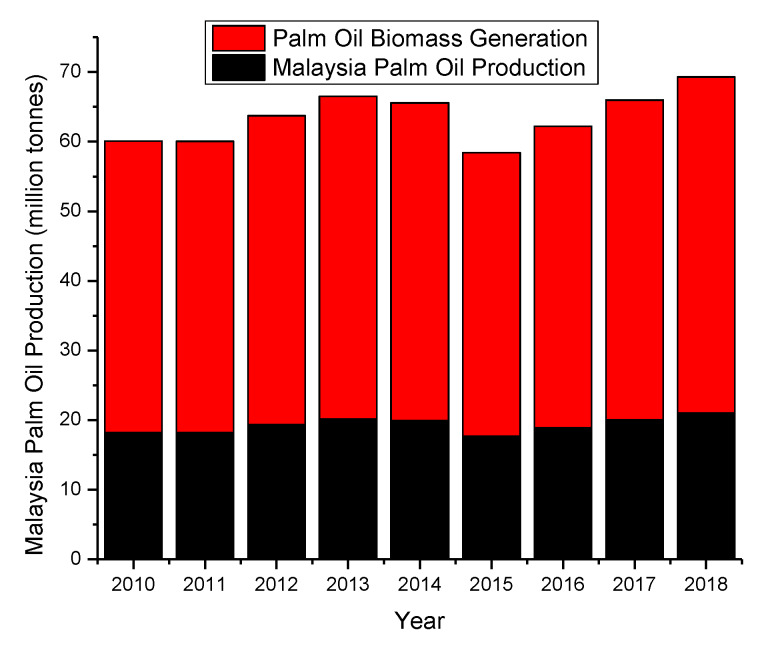
Annual Palm Oil Biomass Waste Generation in Malaysia, adapted from [33,35].

Palm-oil-based biomass is one of the potential resources for sustainable feedstock. A palm oil production plant approximately produces 2.3 tonnes of biomass waste for every 1 ton of palm oil produced [33]. For example, as one of the largest palm oil producers, Malaysia produces around 20 million tonnes of palm oil in 2018 (Figure 5) [35], which means that 45 million tonnes of biomass waste were also generated. Palm oil empty fruit bunch (EFB) is the largest percentage of waste of 43 wt %, which accounts for 19.35 million tonnes of the total biomass waste produced in palm oil production plants [33]. Palm fibers, palm kernels, and palm shells biomass waste were also generated at 30, 13, and 13 wt % respectively. 

In terms of chemical composition, EFB has a high content of cellulose, highlighting its potential as a feedstock source for the biorefinery. Converting EFB biomass waste into fuels and chemicals is thus an economically and environmentally sound proposition. Due to the variability of biomass types, adoption of the available lignocellulosic conversion process and technology is not readily applicable. 

## 4. Potential Chemicals from Biomass

The characteristics of the modern biorefinery are parallel to the petroleum refinery: an abundant raw material consisting primarily of renewable polysaccharides and lignin enters the biorefinery and, through an array of processes, is reacted, fractionated, and converted into a mixture of products, including engine fuels, biochemicals, and direct energy [36]. The imbalance between commodity chemicals and fuels as happened in a petroleum refinery is envisioned to be followed by biorefinery. Chemical products account for only ~5% of petroleum refinery output, while transportation fuels and energy take most of the output [23]. 

Most of the biobased products are the outcome of a direct physical or chemical treatment and processing of biomass, such as cellulose, starch, oil, protein, lignin, and terpene. Through biotechnological processes and methods, feedstock chemicals, such as ethanol, butanol, acetone, lactic acid, and itaconic acid, and amino acids, glutaminic acid, lysine, and tryptophan, can be produced. Of the approximately 170 billion tonnes of biomass produced annually by photosynthesis, less than 0.11% is used for non-food areas, such as chemistry [37], suggesting that there is a huge gap that can be exploited. To enhance the coverage of biorefineries, developments of a vast variety of biobased products in an efficient construction-set system are required to also include particular products that are not accessible in petroleum refineries. There are over 300 possible chemical building blocks that can be derived from biomass [38]. The U.S. Department of Energy applied selection protocols based on the cost of feedstock, estimated processing costs, current volumes, prices, the technical complexity associated with the best processing pathway and the market potential to further screen into 50 top chemical candidates. The next selection criteria are the number of functional groups and potential use as a super chemical commodity. Preference is made for chemicals with more than one functional group. This screening resulted in top candidates and was categorized based on carbon number (C#).

**Figure 6:**
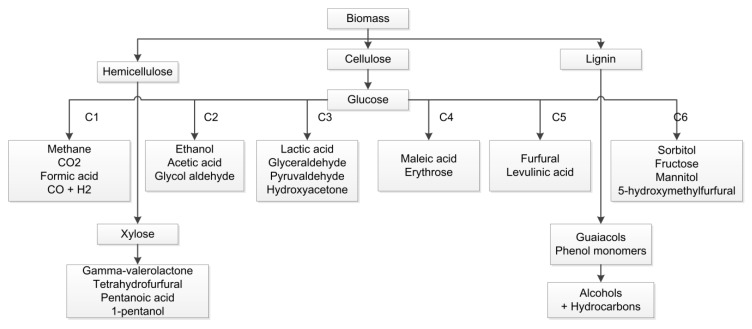
Top Chemical Building Block Candidates from Biomass, adapted from [37].

Kohli [39] considered 5-hydroxylmethylfurfural, phenols, and sugar alcohols as potential platform chemicals for biofuels, biopolymers, and solvent industries (Figure 6). The National Renewable Energy Laboratory (NREL) has conducted a market assessment of bioproducts with near-term potential. The bioproducts-assessed were butadiene, butanediol, ethyl lactate, fatty alcohols, furfural glycerin, isoprene, lactic acid, propanediol, propylene glycol, succinic acid, and xylene [40].

## 5. Optimization of Biorefinery

Biofuels and biochemicals produced through biorefinery are expected to be the backbone of the future sustainable economy. However, there is an impediment to biorefinery facility adoption and implementation. The slow development of biorefineries is caused most critically by the low readiness level of the process and technology. Investment in the construction of a biorefinery is thus seen as a risky business. Most of the existing biorefinery use limited feedstocks and technologies where only a relatively small fraction of materials are converted into high added-value chemicals. A typical biorefinery solely produces a single product such as ethanol or biodiesel. Price wise, products of the biorefinery therefore cannot compete with the petroleum-derived product [41]. 

Process integration and optimization are among the strategies to alleviate the competitiveness of the biorefinery. The benefit of biorefinery integration mainly lays in the diversification of feedstocks and marketable final products. However, the integrated biorefinery still requires continuous improvement and advancement in the areas of feedstock, conversion processes (biochemical, chemical, and thermochemical), and their integration with robust and proven downstream separation processes [42]. The following sub-sections overview the application of mathematical tools in assisting the developments of the biorefinery.

### 5.1. Mixed-Integer Non-Linear Programming

**Figure 7:**
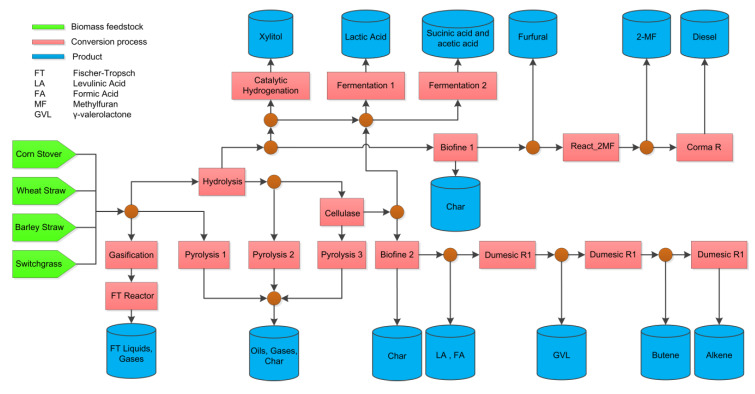
Biorefinery Superstructure with Multiple Feedstocks and Products, adapted from [43].

Multitudes of available biomass feedstocks, processing technologies, and potential products require well-developed formulation to assess and select the most economically, environmentally, and technologically sound biorefinery. Kelloway and Daoutidis [43] formulated a biorefinery superstructure that can produce both fuels and chemicals from different kinds of feedstocks, as illustrated in Figure 7. The biorefinery superstructure involved hydrogenation, gasification, fermentation, pyrolysis, and thermochemical process. The products span from gasoline, diesel, Fischer-Tropsch fuel products, xylitol, lactic acid, succinic acid, furfural, formic acid, and acetic acid.

The optimum biorefinery superstructure is determined by solving the formulation using Mixed–Integer Non-Linear Programming (MINLP) to maximizing NPV (net present value) and carbon efficiency. The optimized carbon efficiency resulted in the preferred biorefinery configuration that produces bio-oils from the pyrolysis reactor, while the base case analysis (only maximizing NPV) favored the choice of biorefinery configuration that produces chemicals such as xylitol, levulinic acid, and formic acid. 

Albarelli et al. [44] demonstrated that process optimization can be used to aid the decision making of two different proposed processing alternatives. A sugarcane-based bioethanol biorefinery integrated with methanol production from sugarcane lignocellulosic residue was compared with the stand-alone bioethanol biorefinery. A thermo-economic model was developed to assess the energy efficiency as well as the economic impact of the integrated process. Two objectives were used, namely the maximization of energy efficiency and the minimization of the investment cost. 

Fattahi and Govindan [45] proposed multi-stage stochastic programming with the Benders decomposition approach to evaluate the sustainable design of biofuel supply chain networks under biomass supply uncertainty and disruption risk. The biomass supply-seasonal fluctuation was modeled as a modified autoregressive. While the disruption risk was related to the storage capacity in the face of biomass yield variation. The yield was modeled as a Bernoulli random variable. Three objectives were used: economic, environmental, and social impacts. A lower and upper bound were set for both environmental and social impacts. The optimum solution from sets of the Pareto optimal solution was obtained using the ε-constraints method. 

Bbosa, Mba-Wright, and Brown [41] conducted a study on the techno-economic analysis of a corn stover ethanol biorefinery integrated with a lignin hydrothermal liquefaction. Corn stover was used because it is the most abundant agricultural residue in the USA and is expected to be the single largest lignocellulosic biomass source in the country. The ethanol plant, as it is known, produces lignin as a waste. Through hydrothermal liquefaction, lignin can be converted into marketable biochemicals. The study was based on an ethanol plant that processed 2000 metric tonnes per day of corn stover to produce 61 MMgal/year of ethanol and different yields of lignin-derived biochemicals. The hydrothermal liquefaction process was set to utilize 80% of the solid/lignin from the recovery section, while the other 20% was used as fuel to generate heat and power for the facility. As a result, the minimum ethanol selling price (MESP) was affected by the yields and market price of the biochemical products. The MESP of the integrated ethanol plant and lignin-derived biochemical was lower than the reference ethanol price (Iowa’s average 2007 ethanol selling price) and much lower than the price of ethanol produced in the stand-alone ethanol plant [41]. 

Another techno-economic analysis was reported by Ou et al. [46] on corn grain and corn stover co-located production plants. The co-locating of an ethanol plant from corn grain and corn stover offers advantages, namely increasing biomass feedstock inventory and reducing production cost. It promotes the commercialization of cellulosic ethanol and improves the competitiveness of corn ethanol to fossil fuel. Corn-based ethanol has been heavily criticized since it causes the food/feed conundrum. The rising price of corn is the result of a competition between food/feed industries and chemical industries. 

Nearly 46% of the US corn crop was used as a feedstock for bioethanol in the year 2011. Despite the heavy use of corn in that year, bioethanol production only equaled 10% of US gasoline production [46]. The study compared the MESP of ethanol produced by the stand-alone Gen 1 plant (corn-grain feedstock), the stand-alone Gen 2 plant (corn-stover feedstock), and the co-located plant (corn-grain and corn-stover feedstock with different mass ratio). The MESP of the co-located ethanol plant was found to be higher than MESP of the Gen-1 plant but lower than the Gen-2 plant. This study, however, did not show the network integration between Gen-1 and Gen-2 plant. Both plants were located in the same location but operated separately.

Zondervan et al. [47] developed a model of a biorefinery that produces multiple products (ethanol, butanol, succinic acid) that were integrated with the supply line of fossil-fuel-based gasoline. The integrated model facilitated the blending of chemical products with gasoline [47]. The resulting superstructure was transformed into MINLP with four different optimization objectives (maximizing profit, minimizing costs, minimizing waste, and minimizing fixed costs) [47]. The different processing steps were formulated as intervals divided over several refining stages containing different operations (splitting, solution, and reaction).

**Figure 8:**
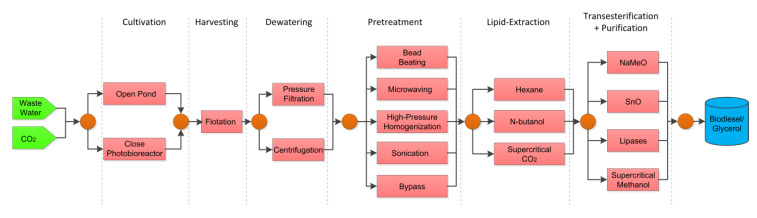
Integrated Algae- and Wheat Straw- Biorefinery that Supplies the CO_2_ and Nutrients in the Wastewater, adapted from [48].

Galanopoulos et al. [48] performed techno-economic analysis through the optimization of an integrated algae biorefinery and wheat straw biorefinery (illustrated in Figure 8) to minimize the total cost of biodiesel production. The wheat straw biorefinery supplies wastewater and CO_2_ as the medium and nutrient to grow the algae. The algae biorefinery utilizes the lipid of algae while the carbohydrate is sent to the wheat straw biorefinery, which produces bioethanol and levulinic acid. The total production cost is chosen as the objective since the development of algae biorefinery has not yet cost-competitive compared to the conventional biorefinery. Henceforth, the possibilities of biorefinery integration and the optimization of algae conversion routes to alleviate its economic feasibility are of interest. The optimization result suggests a reduction of biodiesel total production cost up to 80% if algae biorefinery is integrated with wheat straw biorefinery over a stand-alone algae biorefinery. 

The significant reduction in the total production cost is caused by the unnecessity to procure nitrogen and phosphorus as the nutrient for the algae growth. Furthermore, the integration of algae biorefinery and wheat straw biorefinery provides more choices in treatment and conversion process. The integration opts technology of lower energy consumption, while the stand-alone algae biorefinery opts more energy-intensive processing technology, causing a substantial increase in the operating cost. 

Sy et al. [49] developed a target-oriented robust optimization (TORO) approach for a multi-objective techno-economic and environmental feasibility analysis of an algal-based integrated biorefinery. The TORO resulted in an optimal configuration that was found to be more immune to variation in product demand relative to the configuration resulted from the deterministic optimization. A target value for the objective must be set carefully. Setting the appropriate target value is important. Setting the target value too high or too low or too conservative resulted in a sub-optimal solution. In this study, a robustness index (θ) was introduced in the formulated mathematical representation to measure the highest degree of uncertainty of key model parameters that could be tolerated by a solution before it becomes infeasible. The solution suggested a similar trend of economic criteria (profit) and environmental criteria (carbon footprint), where decreasing profit was followed by decreasing carbon footprint. It might be seen that decreasing profit was caused by the lower production rate and hence lower carbon emission. The optimum integrated algal biorefinery was achieved by a low robustness index.

Pharmaceuticals is another class of products that can be generated through biorefinery. However, the studies on seeking optimum chemical processing technologies for biopharmaceuticals are not as massive as the ones for biofuels and biochemicals. Ng et al. [50] performed a single objective optimization of pharmaceutical production from palm-oil-based biomass through maximization of gross profit. The novelty lays in the fact that pharma-industries are identical with low product yield and high product price in contrary to the fuel refinery. In the low product output, the optimization is forced to find chemical process routes capable of bringing economic gain. In this study, the optimization started with constructing the chemical reaction pathway map (CRPM) consisting of choices of the treatment process and conversion process. The CRPM aided the elimination of chemical process, which was not technologically proven and feasible. The study finds that conversion is a determining parameter for the economic performance of the pharmaceuticals biorefinery. The value of the conversion rate of one product leads the preference to produce other products altogether or the preference of multi-products biorefinery. The sensitivity analysis result reveals that the gross profit of some products stays constant upon the deviation of its respective conversion while the profit of other products is fluctuating. It is not a necessity to invest in the enhancement of the technology to increase the conversion of some products since the increasing yield fails to increase profit. This can be caused by the low market demand of products where the overproduction cannot be absorbed by the market.

Bairamzadeh et al. [51] developed robust possibilistic programming based on mixed-integer linear programming (MILP) for optimization of a lignocellulosic-based bioethanol supply chain under three-uncertain parameters, namely the demand variation of bioethanol, price variation of bioethanol and biomass, and uncertainty in the unit environmental impact coefficients in the environmental objective function. The uncertainty parameters were treated as fuzzy numbers. Multi-objective optimization was performed with coupling social impacts’ objective with the economic and environmental objectives. The social impact was quantified by the number of job creations. The optimization was extended beyond obtaining optimum processing routes and production capacity, but also to determine biomass sourcing, allocation, and location. This study was considered as operational planning with a time horizon of a year divided into months. Interestingly, the production of bioethanol was not constrained to fulfill the market demand, whereas a penalty cost for unmet demand was applied in the objective function of profit maximization. The environmental impact objective function was further expanded into the quantitation of the effect on human health, ecosystem, and resource. 

An interesting study by Singh et al. [52] aimed to capture the dynamics of corn price dues to competition and interactions among biorefineries, among farmers, and between biorefineries and the food market. The commodity price is usually modeled based on historical data without further attempt to understand the extent of corn-user interaction to the price. The accurate prediction of corn price is thus critical since the corn price is the largest cost component to produce bioethanol. An agent-based model was developed, and the resulting corn price’s estimate was returned to the supply chain design problem. The design optimization problem was then solved by a genetic algorithm to seek the optimum location and capacity of each biorefinery in the network under the criteria of maximization of the net present value. 

Giuliano et al. [53] performed process optimization of a biorefinery for the production of levulinic acid, succinic acid, and ethanol to find the optimum process pathway under the criteria of maximizing the net present value or the internal rate of return using a discretization of the MINLP master problem to MILP. The results show that the product selling price, discount rate, and plant scale are significant factors that affected the result of the process optimization. 

López-Díaz et al. [54] employed multi-objective optimization to identify the optimum choice of feedstocks, cultivation sites, the location of biomass processing facilities, and the selection of conversion technologies. The study placed great interest in the optimization of water consumption and discharge during biomass cultivation, pretreatment, and conversion. The biorefinery operation involves a substantial amount of water, whereas the discharge of wastewater into the surroundings affects the water quality. The optimization problem was formulated as MINLP in which the only non-linear terms corresponded to the exponential to consider the economies of scale for the estimation of the capital cost of the biorefinery. Total annual profit was used as the economic-evaluation parameter, whereas the watershed's capacity was set as the environmental constraints. 

### 5.2. Mixed-Integer Linear Fractional Programming

Mixed-integer linear fractional programming (MILFP) has also been studied in chemical process optimization. The MILFP arises when continuous time formulations are used, such as in the maximization of the cyclic profit rate or productivity. This objective takes the form of a ratio between two linear functions with profit as the numerator and the cycle time as the denominator. 

Tong et al. [55] used the unit objective for the optimization of an integrated petroleum refinery and a biorefinery supply chain. In this study, the problem was formulated into MILFP by transforming the objective of minimizing total cost to the minimization of unit cost. The unit objective refers to the total profit or total cost divided by the total amount of the functional unit. A robust optimization technique was employed to solve the optimization problem in which trade-off between robustness and performance became the main issue. The issue arises since the process robustness is often achieved by sacrificing the performance. In this case, parametric and reformulation-linearization approaches are adopted to find optimum solutions. A comparative study was performed between the deterministic programming to minimize the total cost and MILFP to minimize the unit cost. MILFP resulted in higher total cost but lower unit cost compared to the deterministic programming result. As a consequence, a strategic decision was also different between those techniques in terms of preference of technologies and production capacities. The reduction of complexity through transformation into MILP or the use of stochastic optimization strategies such as the Monte Carlo can produce a good solution. However, there is a possibility that the process misses better points to be evaluated in the solution domain. 

Salas et al. [56] proposed a stochastic metaheuristics optimization method to optimize a lignocellulosic-based biorefinery under operational level uncertainties. In the proposed method, the operating points were intelligently searched rather than randomly searched as in the Monte-Carlo simulation. 

Ng and Maravelias [57] constructed a MILP for the design and operational planning of the cellulosic biofuel supply chain. An approximation and linearization approach for the calculation of shipment and transportation distance was adopted to obtain the linear model. Bairamzadeh, Saidi-Mehrabad, and Pishvaee [11] employed robust optimization programming to solve the MILP model of the lignocellulosic-based bioethanol supply chain. The model was developed to handle disparate types of uncertainty (randomness, epistemic, and deep uncertainty). There were three uncertain parameters, namely conversion rates, biomass yield, and demand. Uncertainty in the process was manifested in terms of unprecise conversion rates. Probability-based scenarios were defined to express this particular uncertainty. Biomass yield was expressed as a fuzzy number, while the demand was assumed to vary in a known interval. 

### 5.3. Possibilistic Programming

Babazadeh [58] developed a robust optimization method for the optimization of biomass to the bioenergy system under deep uncertainty. Deep uncertainty arises from the lack of availability of historical data and the limited information on the input parameters, which means that possibility and probability distribution are impossible to construct. The deep uncertainty of parameters was modeled using a polyhedral uncertainty set. 

Mousavi Ahranjani et al. [59] proposed robust possibilistic programming (MORPP) for the multi-objective optimization of a multiperiod multi feedstock lignocellulosic biofuel supply chain network under epistemic uncertainty. The robust possibilistic programming was developed to overcome the drawbacks of stochastics programming. Stochastics programming requires a large number of scenarios. Furthermore, the size of the stochastics programming is further enlarged by the increasing number of parameters and probability. In the possibilistic programming, the average value of uncertain parameters is used to obtain the solutions without control of deviations from the expected or mean value of the objective function. The model in this study was developed to determine the optimal location, capacity, conversion technology, transportation modes, material flow, and production planning of biorefineries. The objectives used in this study were economic, environmental, and social aspects. 

A multi-objective robust possibilistic programming was again adopted by [15] to conduct the optimization of switchgrass-based bioethanol supply chain network under the epistemic uncertainty. Three conflicting objectives were modeled, namely the economic, environmental, and social impacts. The proposed approach was able to maximize the mean value of supply chain performance and control the optimality as well as feasibility robustness. It outperformed the deterministic optimization in terms of average and standard deviation measures. 

Rabbani et al. [60] used multicriteria decision-making methods, TOPSIS, and augmented ε-constraint, to find the Pareto optimal solution of the switchgrass-based bioenergy production system. It was modeled as MILP with three conflicting objectives and was solved via two-stage algorithms. In the first stage, the ε-constraint was used to obtain sets of Pareto optimal solutions. The next step was to use TOPSIS to rank the solutions with respect to the weight for each objective function. TOPSIS works by determining the best alternative that has the shortest distance from positive-ideal solution and the longest distance from the negative-solution. The planning horizon in this study was short with an extent up to 3 years divided into twelve periods in accordance with the harvesting periods. 

Santibañez-Aguilar et al. [61] performed multi-objective optimization for biorefinery supply chain with total annual profit and Eco-indicator99 as the economic and environmental objective, respectively. The uncertainty scenario based on biomass prices was generated using Latin Hypercube. Each MILP model was then deterministically solved using the Monte-Carlo method. 

The model and approach to optimization problems should guarantee optimality robustness and feasibility robustness of the solution. Optimality robustness is attained when the objective function value of the solution vector remains close to the optimal value or have a minimum deviation from the optimal value. Feasibility robustness is achieved when the solution vector stays feasible for almost all possible values of uncertain parameters. 

There are three types of uncertainty prevailing in the process optimization of biorefinery: randomness, epistemic, and deep uncertainty. The process optimization problem with the randomness type of uncertainty can be catered by stochastic programming, epistemic by possibilistic programming, and deep uncertainty by robust convex programming. Realistically, all the uncertainties can present in the process optimization of biorefinery.

## 6. Optimization of Petroleum Refinery

**Figure 9:**
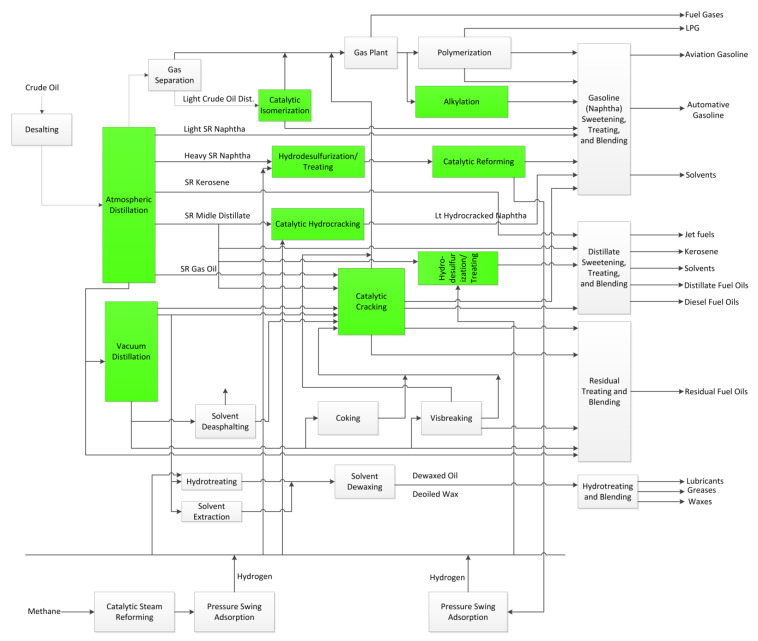
Process Flow Diagram of Modern Petroleum Refinery, adapted from [64].

Petroleum refinery configuration, illustrated in Figure 9**,** comprises of crude distillation units (CDU), catalytic reforming units (CRU), delayed coking unit (DCU), fluid catalytic cracking units (FCC), hydrotreating units (HTU), hydrocracking units (HCU), gasoline blender (GB), and diesel blender (DB) [62]. CDU fractionates the crude oil into heavier and lighter fractions. The fractions are gas, light straight-run naphtha, heavy straight-run naphtha, straight-run kerosene, straight-run middle distillate, straight-run gas oil, and vacuum residue [63]. The vacuum residue is further fractionated in the vacuum distillation unit. The heavy straight-run naphtha with low octane rating is treated in the catalytic reforming unit to produce higher octane liquids. The heavier fractions from the vacuum distillation unit are treated in the DCU to generate a higher quality of fuel oil [62]. The straight-run middle distillate, straight-run gas oil, light vacuum distillate, and heavy vacuum distillate are treated in the FCC units. The fractions are broken down and rearranged into lighter molecules to increase the quality and the quantity of the products. The straight-run middle distillate can also be treated with hydrogen in the HTU to remove the sulfur content. The effluent of the HT is later be blended to produce diesel fuel oils. The HCU cracks the straight-run middle distillate under hydrogen feed to generate gasoline and kerosene. 

The vast options of available processes in petroleum refining lead to a very large number of refinery configurations, such that designing a petroleum refinery involved 200 ready-made heuristic plot plans [65]. It is certainly a daunting task to find the optimum design. The complexity is further escalated since refineries are subject to numerous uncertainties during operation, namely the fluctuation of crude oil price, crude oil availability, and changing the demand level [66]. Shah et al. [67] identified three major uncertainties in the refinery production planning, namely (i) market demand for products, (ii) the prices of crude oil and the saleable product, and (iii) the production yields of crude oil in the primary crude distillation unit. In terms of saleable products, gasoline and diesel make up about 60–70% of the revenue of a refinery [16]. 

Most of the previous studies of oil refinery planning were deterministic in which the strategies resulted from the deterministic approach show a lack of robustness or become infeasible upon the realization of uncertainty parameters [66]. Ribas et al. [68] investigated the impact of uncertainty on investment decisions in the integrated oil supply chain using stochastic and robust programming. The research attempted to handle the planning problem at the strategic level where the prior studies focused to treat refinery planning problems at the tactical and operational levels. Three different models were developed, namely a two-stage stochastic model with fixed recourse, a min–max regret model, and a max–min model.

Al-Qahtani et al. [69] studied the optimization of multisite refinery networks under the uncertainty of the raw material price, product price, and demand through stochastic programming and robust optimization. MILP was formulated for the stochastic model, while the MINLP was formulated for the robust optimization with a single objective of minimization of the total cost. The non-linearity arose from the modeling the risk components. 

A particular aspect of petroleum refinery operation that has gained increasing interest is the hydrogen networks. The refinery hydrogen networks, in general, comprise of hydrogen consuming and producing units, hydrogen purification units, compressors, and pipeline systems. The pipeline system in the refinery hydrogen networks facilitates the distribution of low-pressure gas and high-pressure gas. The gas in the lower pressure pipeline system can be compressed and the gas in the high-pressure pipeline system can be purged [70]. Hydrogen is needed for the HTU, HCU, FCC, and isomerization unit. In a refinery, hydrogen is generated from the catalytic reforming unit and the hydrogen plant and is distributed through a complex distribution network for hydrodesulfurization and hydro-treating processes [71]. The high purity hydrogen is produced through steam reforming or partial oxidation of light hydrocarbon fractions, such as refinery-off gas or light naphtha [72]. The lower purity of hydrogen is generated as the byproduct of the cyclization and dehydrogenation of hydrocarbon to increase the aromatics content and the octane number of naphtha products in the catalytic reforming unit [72]. As for current practice, the refinery is required to produce fuel with lower aromatic content; thus, hydrogen generated as a by-product in those processes is also lower [72,73]. Moreover, the petroleum refinery must process crude oil with a higher content of sulfur and nitrogen [74]. Consequently, the processing capacity of hydrocracking and hydro-treating units in the refinery will be changing over time. 

The production and distribution systems of hydrogen are required to anticipate the changes in hydrogen demand and requirement to ensure a sustainable operation. In the production of hydrogen, there is a tradeoff between production load, efficiency, and emission. A study of a refinery in China reveals that the low hydrogen production load decreases the exergy efficiency but increases the CO_2_ emission [75]. The need for the optimum scheduling of hydrogen production and distribution relies on the gain of lower refinery operating costs, safer operation, and lower environmental impact. As stringent environmental regulations and policy require the refinery to produce cleaner fuel with lower sulfur and gasoline of lower aromatics content, the refinery will likely to build new hydrogen plants. The capital costs of hydrogen production units are high. It was estimated to be equivalent to more than one-third of the capital costs of hydrocarbon conversion units for upgrading heavy petroleum fractions [72]. It is in the view of a sustainable process that the management of hydrogen generation and consumption should be regarded as a critical engineering aspect in the refinery [72]. 

The hydrogen network integration is at the forefront approach for refinery hydrogen management. Many integration methods have been studied and can be categorized into the pinch based methodologies and mathematical programming techniques [73]. Research on hydrogen refinery networks’ optimization has shifted from single-period optimization to multi-period optimization to account for the uncertainty factors present in the operation of hydrogen refinery networks, such as a change in the operating parameters. Recent studies also consider a multiple-impurity system where the stream of the hydrogen refinery networks is not limited to only a mixture of hydrogen and methane but also H_2_S and CO [62]. The multiple impurity system better represents the real hydrogen refinery network.

Petroleum refinery consumes a high amount of energy. Energy saving leads to lower operational cost, hence more profit can be obtained. Most of the process optimization only considers material transfer (within the process) to achieve optimum use of materials. While the energy transfer that might be conceived is not accounted. This will overestimate the utility cost. Chen et al. [76] performed simultaneous process optimization and heat integration for methanol production process. The result was compared with the optimized base case without heat integration and sequential optimization and heat integration. In the sequential optimization, heat integration was performed after the process was optimized, while the simultaneous approach was solved numerous times depending on the number of iterations. The simultaneous approach resulted in decreased of utility consumption and higher profit. Tovar-Facio et al. [77] performed the process optimization of a water network for oil refineries integrated with an electrocoagulation treatment system. The study resulted in a reduction in fresh-water consumption and waste generation compares to the water network without electrocoagulation. The process optimization also showed that the integration of electrocoagulation substantially reduced the total annualized cost. Those case studies demonstrate that the optimum use of materials and energy in chemical processing facilities can be determined through mathematical programming-based process optimization.

## 7. Sustainability Parameters in Multi-Objective Optimization

Sustainability is the ultimate goal for the development of the economy and future biorefinery systems. It has become the objective of many studies on the process optimization of the production system (see Table 1). The sustainability concept comprises not only economic and environmental aspects but also has widened out into the areas of social, safety, and health. The impression of the biorefinery system as sustainable due to the renewability of the biomass sources has been refuted given that sustainability is not founded solely on renewability or the environmental dimension. All dimensions of sustainability shall be counted in the development of future biorefineries. 

The consideration of sustainability parameters in the early stage of biorefinery design could have a significant impact on improving the overall performance and consequently results in alternative design options. There are several ways to analyze the economy of a chemical process facility: the minimization of cost, maximization of profit, and the maximization of net present value, etc. The minimization of cost and maximization of profit are suitable for the optimization of the existing and already running facilities, while the maximization of net present value is suitable for optimization of the proposed design of a new facility. In a certain case, the maximization of net present value suggests omitting some process. Thus, it helps the decision maker on making the right investment [62].

Global warming and environmental pollution, the negative impacts of industrialization, have made people realize that a more sustainable practice of industrialization should be embraced. Therefore, the incorporation of environmental objectives alongside economical objective in the process optimization of a chemical plant is of paramount important. The common factors of addressing sustainability include life-cycle assessment, carbon dioxide utilization, process safety and social impact, all of which are addressed in the following section. The inclusion of sustainability parameters during system optimization requires multi-objective routines. Sustainability factors, together with the already complex nature of the refinery system, make system optimization challenging.

### 7.1. Life-Cycle Assessment (LCA) 

Life-Cycle Assessment (LCA) is regarded as the most scientifically reliable method to assess the environmental impact of a product or process. It is an objective process for evaluating the environmental burdens associated with a product, process, or activity [78]. LCA begins with the establishment of analysis boundaries and followed by inventory analysis and impact assessment. The inventory analysis quantifies the materials and energy consumed and emissions and waste generated during the operation, while the impact assessment involves the quantification of global warming potential (GWP) and damage impact. 

Wang et al. [78] performed the optimization of hydrocarbon biorefinery via gasification with the objective criteria of LCA for the integrated life cycle assessment and techno-economic analysis. The study aimed to find the Pareto-optimal of minimizing GWP and maximizing NPV. Simapro and Ecoinvent software are frequently used to perform LCA analysis of a biorefinery system [79,80].

### 7.2. Process Safety

Inherent risk associated with the operation of chemical process plants should be thoroughly considered during the design of a chemical process plant. The capability to quantify and to rank risk helps in determining the risk appetites and the corresponding safety measures. Typically, the safety aspects are assessed once the design of the chemical process plant has been completed and the operating parameters have been specified [81]. 

Hazard and operability (HAZOP) and quantitative risk assessment (QRA) are the common methodologies to evaluate the safety aspects. However, both methods are not suitable to assess safety in the conceptual phase of plant design. HAZOP requires detailed information on the process that is only available once the design is completed. However, QRA becomes inconvenient for the safety analysis of complex facilities since it only estimates the failures and consequences of a piece of equipment or system of a few units with a probabilistic approach [81]. 

The assessment of inherent safety in the design phase of a chemical process plant requires metrics that allow direct calculation with limited information. The material and process factors are typically incorporated into the metrics. There are two of such metrics: (i) Dow fire and explosion index (F&EI) (ii) fire and explosion damage index (FEDI). The FEDI is developed to overcome the limitation of F&EI in which the latter treats material factors as independent to process operating conditions. The estimation of FEDI comprises the classification of units based on the purpose, the evaluation of energy factor dues to chemical properties and operating conditions, the assignment of penalty, and the estimation of potential damage [81]. 

A different approach was reported by El-Halwagi et al. [82]. in their study of the multi-objective optimization of biorefineries with economic and safety objectives. The economic objective was to minimize the total annual cost associated with the cost of purchasing and transporting the feedstocks, the processing cost in the central facilities, the transportation cost of biofuels from central facilities to the users, the processing cost of the biofuels in the industrial facilities, and the capital costs for the biofuel production. As for the safety objective, the study proposed an approach that relates the number of fatalities per year and the quantity of biofuels produced/bought per year. The probit approach was used to determine the number of fatalities where it is used in the quantitative risk analysis to predict the number of acute fatalities caused by an accident.

### 7.3. Social Impact

Social impacts are associated with social justice and the rights of stakeholders, including employees, customers, and local communities [83]. The opted indicators to justify social impacts can be varied. Regarding the transformation of land usage, the food footprint can be used to measure the area that is converted from food production to energy production. The number of jobs created is the widely used social impact indicator since it is a real social benefits indicator. However, the maximization of job creation is not always the right objective. In a decreasing population, the optimization model might involve the minimization of job creation. The social impacts should be considered during the process of selecting the optimum biorefinery location. 

Cambero and Sowlati [84] quantified the overall social benefits of jobs created by implementing the weighted sum of all the new jobs created across the supply chain. The assigned weight is based on the preferability of creating jobs in each location. 

## 8. Integration of Petroleum Refinery and Biorefinery

Bio-oil resulting from the pyrolysis of biomass has the potential to be co-processed with petroleum feeds in the existing refinery unit to produce biofuel [85]. It seems to be the most probable intermediate product of biorefinery for linkage with the petroleum refinery. In terms of economics, the retrofitting cost of the petroleum refinery to co-process bio-oil is considered economically viable; the integration of biorefinery into existing petroleum refinery results in considerable capital savings on the biofuel production facilities construction that leads to higher competitiveness of the biofuel [62]. 

Research has been conducted on the lab-scale and pilot-scale of co-processing bio-oil and petroleum feeds in the FCC unit. The parameters being investigated are the type of bio-oil (raw bio-oil, upgraded bio-oil, hydrodeoxygenated bio-oil), the type of catalysts, the ratio of bio-oil to vacuum gas oil, etc. However, there are few studies on the optimization of integrated petroleum refinery and biorefinery. 

The integration of the petroleum refinery and the petrochemical facility has also been studied. Al-Qahtani et al. [86] studied the optimization of petrochemical network design in which the uncertainty parameter was accounted to accommodate the changing of process yield, raw material cost, product prices, and product demand. The problem was formulated as a two-stage stochastic MINLP where the non-linearity manifests from modeling the risk components. The results show that the variations in process yield and product demand more dominantly affect the sensitivity of the petrochemical network. 

Al-Qahtani and Elkamel [87] expanded the study to integrate and to optimize multisite refinery and petrochemical systems. MILP was developed with the objectives of minimizing the annualized cost over the set time horizon among the refineries and maximizing the added value of the petrochemical network. The process integration, utility integration (heat/steam/hydrogen/power), and fuel gas upgrading were among the aspects modeled in this study. The study was in a certain way motivated by the previous findings in which a significant energy consumption reduction (up to 60%) was achievable through the integration of energy sources and sinks of the steam cracking with other industrial processes. Furthermore, the integration of a gas turbine through gas-exhaust recovery between petrochemical units and ammonia plants has the potential to reduce energy usage by up to 10%. The integration of the hydrogen network is becoming crucial due to a stringent environmental regulation requiring refineries to lower the sulfur content in the fuels that are produced. Consequently, the hydrogen consumption increases to achieve deeper desulfurization.

The developed formulation Al-Qahtani and Elkamel [87] was implemented to study the integration of petrochemical complex producing poly(vinyl chloride) (PVC) and three complex refineries. The refineries complex produced liquefied petroleum gas, light naphtha, two grades of gasoline, jet fuel, military jet fuel, gas oil, diesel fuel, heating fuel oil, and petroleum coke. The feedstock of the PVC petrochemical complex is the light naphtha and gas oil produced from the refineries. The optimization was performed separately for refineries complex and integrated refineries complex-petrochemical complex. The total annual cost for the refineries complex was found to be higher than the total annual cost of the integrated refineries complex and PVC complex. The model also suggested that gas oil, an intermediate stream of the refineries, is the preferred feedstock for the ethylene cracker of PVC complex against the more commonly used light naphtha. The light naphtha was better allocated for maximum gasoline production. The integration of the petroleum refineries complex and petrochemical complex increased the capacity utilization up to 100% of the gas oil desulfurization unit for all refineries. 

Al-Sharrah et al. [88,89] developed multi-linear programming for the optimization of petrochemical facilities in Kuwait. The study aimed to optimize the petrochemical network under two criteria objectives: the maximization of profit and the minimization of environmental impact. The environmental objective was quantified by the health index of the chemicals set by the National Fire and Protection Agency. 

## 9. Conclusions and Perspectives

Liquid fuel is the largest product consumed from among a wide array of petroleum-refinery products. As society becomes sophisticated and more environmentally conscious, the switch of petroleum-based to renewable feedstock-based biorefinery is inevitable. The first-generation biomass-based biorefinery, which produces bioethanol and biodiesel, has been adopted. However, the use of edible biomass sources has intensified the food and feed vs. the fuel industries' contestation. In the extended term, the first generation of biomass will no longer be sustainable in terms of economic potential. The second-generation biomass and third-generation biomass are attracting great interest as the substitute for the first-generation biomass. 

The decision to adopt biorefinery should be taken with great care since there are a plethora of options for processes and technologies. On top of maximizing profit, sustainability factors shall be considered, which makes the optimization process challenging, which then requires mathematical programming as a powerful tool. The uncertainty of internal and external variables are parameters that affect the sustainability criteria. An example of internal parameter uncertainty is yield or conversion, both of which depend on the operating variables. External uncertainty arises from the variation in demand level and price. To add to this complexity of the decision-making process, the biorefinery is expected to fulfill all the multi-criteria of sustainability, namely economic sustainability, environmental sustainability, safety, and social impacts. 

The indicators for economic and environmental sustainability are already established and adopted. The NPV and cost are the commonly used economic indicator. For the environmental impact, global warming potential and CO_2_-equivalent emission are intensively used. The safety aspect is of paramount importance but is still rarely used as the objective to synthesize a sustainable system. Nevertheless, it will not be feasible to establish biorefinery that results in high economic benefit, but, at the same time, it has a high risk of explosion. In this particular topic, the FEDI or F&EI can be used to measure the safety level of industrial facilities. However, a standard should be established regarding the FEDI or F&EI value that is still universally acceptable. Furthermore, a correlation between the FEDI or F&EI and the required investment for safety improvement should be established. Thus, this safety objective can be monetized. The multi-objective optimization will be more practical if all the objectives are in the same unit. It should be noted that the economical objective is money related and the environmental objective is related to emission level. 

Only a few studies have included the competition factor between agents. The agents in the biorefinery supply chain includes material suppliers, biomass producers, biorefineries, and markets. This competition is impacting the cost of materials and the price of products. For example, the cost of biomass will be lower if there are more biomass producers. The same goes for the price of the product; the price will be lower if there are more biorefineries and will increase if there are more consumers. 

For the environmental impact, CO_2_-equivalent emission can be monetized by the implementation of a CO_2_-penalty cost. For the social impact, the FEDI or F&EI should be monetized by calculating the required investment for safety infrastructure to improve the safety level. In this case, the monetization of the environmental impact and safety impact should be established to be coherent with the unit of economic impacts. 

The biorefinery prospect in countries such as Malaysia, which generate an abundance of palm oil biomass waste, needs to be examined. It should comprise of techno-economic, environmental impact and social impact analyses. Albeit its prospective potential, the willingness of industries to adopt biorefinery depends on the economic potential. The techno-economic analysis should not only consider the internal parameters related to the chemical process, but also the external parameters which are characteristically uncertain. The decision-making process to adopt the biorefinery based on overly deterministic assumption leads to sub-optimal solution. This sub-optimality leads to a narrow capability to accommodate the changing of the uncertainties. Mathematical programming-based process optimization is a powerful technique to address the challenge. However, process optimization requires comprehensive mathematical formulation that can represent the model as accurate as possible. The other prominent challenge discussed in this manuscript is the uncertain parameters involved in the mathematical model. Without fulfilling these two requirements, process optimization will lack the information required to aid the decision-making process. 

## Figures and Tables

**Table 1:** Summary of Biorefinery Process Optimization Case Studies.

No	Case Study	Sustainability Parameter				Uncertainty	Methods	Ref
		Economy	Environment	Safety	Social Impact			
1	Biomass to bioenergy	Total cost	CO_2_-equivalent	-	-	Deep uncertainty	Robust Optimization	[58]
2	Multi feedstock lignocellulosic-based bioethanol	Total cost	GHG emission	-	Job opportunities	Epistemic uncertainty	Robust Possibilistic Programming (MILP)	[59]
3	Switch-grass based bioethanol biorefinery	Total cost	Environmental impact (Point-based)	-	Employment and economic development indicators	Epistemic uncertainty	Robust Possibilistic Programming (MILP)	[15]
4	Lignocellulosic-based bioethanol supply chain	Total cost	-	-	-	Combined-randomness, epistemic, and deep uncertainty.	Robust optimization (MILP)	[11]
5	Switch-grass based bioenergy production	Total cost	GHG emission	-	Jobs creation	-	MILP	[60]
6	Sugarcane-based bioethanol	Investment cost	-	-	-	-	Genetic algorithm (MINLP)	[44]
7	Cellulosic biofuel supply chain	Total annual cost	-	-	-	-	MILP	[57]
8	Biorefinery supply chain	Net annual profit	Eco-indicator99	-	-	Stochastic scenarios, Latin Hypercube, Monte-Carlo	Deterministic programming	[61]

## References

[1] Kamm B., Kamm M. (2004). Biorefinery – Systems. Chem. Biochem. Eng. Q.

[2] Kamm B., Kamm M.J.A.M. (2004). Principles of biorefineries. Appl. Microbiol. Biotechnol..

[3] Lee X.J., Ong H.C., Gan Y.Y., Chen W.-H., Mahlia T.M.I. (2020). State of art review on conventional and advanced pyrolysis of macroalgae and microalgae for biochar, bio-oil and bio-syngas production. Energy Convers. Manag..

[4] Wibisono Y., Agung Nugroho W., Akbar Devianto L., Adi Sulianto A., Roil Bilad M. (2019). Microalgae in Food-Energy-Water Nexus: A Review on Progress of Forward Osmosis Applications. Membranes.

[5] Abdurakhman Y.B., Putra Z.A., Bilad M.R. (2017). Process simulation and economic analysis of biodiesel production from waste cooking oil with membrane bioreactor. Aip Conf. Proc..

[6] Bhullar L., Adi Putra Z. (2017). Process Design and Modelling of the Production of Butyl Cellosolve Acetate and EO-3 Phosphate Ester. Indones. J. Sci. Technol..

[7] Nayaggy M., Adi Putra Z. (2019). Process Simulation on Fast Pyrolysis of Palm Kernel Shell for Production of Fuel. Indones. J. Sci. Technol..

[8] Zainal S., Daud W., Adi Putra Z., Nor N. (2018). Integrated constraints optimization for surface and sub-surface towards CAPEX free maximizing production. Iop Conf. Ser. Mater. Sci. Eng..

[9] Budiman Abdurakhman Y., Adi Putra Z., Bilad M.R., Md Nordin N.A.H., Wirzal M.D.H. (2018). Techno-economic analysis of biodiesel production process from waste cooking oil using catalytic membrane reactor and realistic feed composition. Chem. Eng. Res. Des..

[10] Alias A.F., Putra Z.A., Bilad M.R., Wirzal M.D.H., Nordin N.A.H.M. (2020). Simulation of co-processing bio-oil and vgo in fluid catalytic cracking units. Platf. A J. Eng..

[11] Bairamzadeh S., Saidi-Mehrabad M., Pishvaee M.S. (2018). Modelling different types of uncertainty in biofuel supply network design and planning: A robust optimization approach. Renew. Energy.

[12] Al-Othman W.B.E., Lababidi H.M.S., Alatiqi I.M., Al-Shayji K. (2008). Supply chain optimization of petroleum organization under uncertainty in market demands and prices. Eur. J. Oper. Res..

[13] Gargalo C.L., Carvalho A., Gernaey K.V., Sin G. (2017). Optimal Design and Planning of Glycerol-Based Biorefinery Supply Chains under Uncertainty. Ind. Eng. Chem. Res..

[14] Chen Y., Yuan Z., Chen B. (2018). Process optimization with consideration of uncertainties—An overview. Chin. J. Chem. Eng..

[15] Ghaderi H., Moini A., Pishvaee M.S. (2018). A multi-objective robust possibilistic programming approach to sustainable switchgrass-based bioethanol supply chain network design. J. Clean. Prod..

[16] Oliveira F., Grossmann I.E., Hamacher S. (2014). Accelerating Benders stochastic decomposition for the optimization under uncertainty of the petroleum product supply chain. Comput. Oper. Res..

[17] (2018). OPEC Annual Statistical Bulletin.

[18] Crude Oil Production. https://data.oecd.org/energy/crude-oil-production.htm.

[19] Huynh T.M., Armbruster U., Atia H., Bentrup U., Phan B.M.Q., Eckelt R., Nguyen L.H., Nguyen D.A., Martin A. (2016). Upgrading of bio-oil and subsequent co-processing under FCC conditions for fuel production. React. Chem. Eng..

[20] Ong H.C., Milano J., Silitonga A.S., Hassan M.H., Shamsuddin A.H., Wang C.T., Mahlia T.M.I., Siswantoro J., Kusumo F., Sutrisno J. (2019). Biodiesel production from Calophyllum inophyllum-Ceiba pentandra oil mixture: Optimization and characterization. J. Clean. Prod..

[21] Silitonga A., Shamsuddin A., Mahlia T., Milano J., Kusumo F., Siswantoro J., Dharma S., Sebayang A., Masjuki H., Ong H.C. (2020). Biodiesel synthesis from Ceiba pentandra oil by microwave irradiation-assisted transesterification: ELM modeling and optimization. Renew. Energy.

[22] Goh B.H.H., Ong H.C., Cheah M.Y., Chen W.-H., Yu K.L., Mahlia T.M.I. (2019). Sustainability of direct biodiesel synthesis from microalgae biomass: A critical review. Renew. Sustain. Energy Rev..

[23] Ragauskas A.J., Williams C.K., Davison B.H., Britovsek G., Cairney J., Eckert C.A., Frederick W.J., Hallett J.P., Leak D.J., Liotta C.L. (2006). The path forward for biofuels and biomaterials. Science.

[24] Silitonga A.S., Atabani A.E., Mahlia T.M.I., Masjuki H.H., Badruddin I.A., Mekhilef S. (2011). A review on prospect of Jatropha curcas for biodiesel in Indonesia. Renew. Sustain. Energy Rev..

[25] Silitonga A.S., Mahlia T.M.I., Kusumo F., Dharma S., Sebayang A.H., Sembiring R.W., Shamsuddin A.H. (2019). Intensification of Reutealis trisperma biodiesel production using infrared radiation: Simulation, optimisation and validation. Renew. Energy.

[26] Commission E. Renewable Energy Directive. https://ec.europa.eu/energy/en/topics/renewable-energy/renewable-energy-directive.

[27] Spot Prices for Crude Oil and Petroleum Products. https://www.eia.gov/dnav/pet/hist/LeafHandler.ashx?n=PET&s=RWTC&f=A.

[28] Wallace S.W., Ziemba W.T. (2005). Applications of Stochastic Programming.

[29] Mahlia T.M.I., Syazmi Z., Mofijur M., Abas A.E.P., Bilad M.R., Ong H.C., Silitonga A.S. (2020). Patent landscape review on biodiesel production: Technology updates. Renew. Sustain. Energy Rev..

[30] Silitonga A.S., Masjuki H.H., Mahlia T.M.I., Ong H.C., Chong W.T., Boosroh M.H. (2013). Overview properties of biodiesel diesel blends from edible and non-edible feedstock. Renew. Sustain. Energy Rev..

[31] Yuan Z., Chen B., Gani R. (2013). Applications of process synthesis: Moving from conventional chemical processes towards biorefinery processes. Comput. Chem. Eng..

[32] Crocker M. (2010). Thermochemical Conversion of Biomass to Liquid Fuels and Chemicals.

[33] Chang S.H. (2014). An overview of empty fruit bunch from oil palm as feedstock for bio-oil production. Biomass Bioenergy.

[34] Aresta M., Dibenedetto A., Dumeignil F. (2012). Biorefinery: From Biomass to Chemicals and Fuels.

[35] Malaysian Palm Oil Board Production of Crude Palm Oil for the Month of December 2017. http://bepi.mpob.gov.my/index.php/en/statistics/production/177-production-2017/792-production-of-crude-oil-palm-2017.html.

[36] Ong H.C., Masjuki H.H., Mahlia T.M.I., Silitonga A.S., Chong W.T., Yusaf T. (2014). Engine performance and emissions using Jatropha curcas, Ceiba pentandra and Calophyllum inophyllum biodiesel in a CI diesel engine. Energy.

[37] Kohli K., Prajapati R., Sharma B.K. (2019). Bio-Based Chemicals from Renewable Biomass for Integrated Biorefineries. Energies.

[38] Zoebelein H. (2001). Dictionary of Renewable Resources.

[39] Werpy G.P. (2004). Top Value Added Chemicals from Biomass: Volume I—Results of Screening for Potential Candidates from Sugars and Synthesis Gas. DOE/GO-102004-1992.

[40] Biddy M.J., Scarlata C., Kinchin C. (2016). Chemicals from Biomass: A Market Assessment of Bioproducts with Near-Term Potential.

[41] Bbosa D., Mba-Wright M., Brown R.C. (2018). More than ethanol: A techno-economic analysis of a corn stover-ethanol biorefinery integrated with a hydrothermal liquefaction process to convert lignin into biochemicals. Biofuels Bioprod. Biorefin..

[42] Asadi E., Habibi F., Nickel S., Sahebi H. (2018). A bi-objective stochastic location-inventory-routing model for microalgae-based biofuel supply chain. Appl. Energy.

[43] Kelloway A., Daoutidis P. (2014). Process Synthesis of Biorefineries: Optimization of Biomass Conversion to Fuels and Chemicals. Ind. Eng. Chem. Res..

[44] Albarelli J.Q., Onorati S., Caliandro P., Peduzzi E., Meireles M.A.A., Marechal F., Ensinas A.V. (2017). Multi-objective optimization of a sugarcane biorefinery for integrated ethanol and methanol production. Energy.

[45] Fattahi M., Govindan K. (2018). A multi-stage stochastic program for the sustainable design of biofuel supply chain networks under biomass supply uncertainty and disruption risk: A real-life case study. Transp. Res. Part E Logist. Transp. Rev..

[46] Ou L., Brown T.R., Thilakaratne R., Hu G., Brown R.C. (2014). Techno-economic analysis of co-located corn grain and corn stover ethanol plants. Biofuels Bioprod. Biorefin..

[47] Zondervan E., Nawaz M., de Haan A.B., Woodley J.M., Gani R. (2011). Optimal design of a multi-product biorefinery system. Comput. Chem. Eng..

[48] Galanopoulos C., Kenkel P., Zondervan E. (2019). Superstructure optimization of an integrated algae biorefinery. Comput. Chem. Eng..

[49] Sy C.L., Ubando A.T., Aviso K.B., Tan R.R. (2018). Multi-objective target oriented robust optimization for the design of an integrated biorefinery. J. Clean. Prod..

[50] Ng S.Y., Ong S.Y., Ng Y.Y., Liew A.H.B., Ng D.K.S., Chemmangattuvalappil N.G. (2017). Optimal Design and Synthesis of Sustainable Integrated Biorefinery for Pharmaceutical Products from Palm-Based Biomass. Process Integr. Optim. Sustain..

[51] Bairamzadeh S., Pishvaee M.S., Saidi-Mehrabad M. (2016). Multiobjective Robust Possibilistic Programming Approach to Sustainable Bioethanol Supply Chain Design under Multiple Uncertainties. Ind. Eng. Chem. Res..

[52] Singh A., Chu Y., You F. (2014). Biorefinery Supply Chain Network Design under Competitive Feedstock Markets: An Agent-Based Simulation and Optimization Approach. Ind. Eng. Chem. Res..

[53] Giuliano A., Cerulli R., Poletto M., Raiconi G., Barletta D. (2016). Process Pathways Optimization for a Lignocellulosic Biorefinery Producing Levulinic Acid, Succinic Acid, and Ethanol. Ind. Eng. Chem. Res..

[54] López-Díaz D.C., Lira-Barragán L.F., Rubio-Castro E., Ponce-Ortega J.M., El-Halwagi M.M. (2017). Optimal location of biorefineries considering sustainable integration with the environment. Renew. Energy.

[55] Tong K., You F., Rong G. (2014). Robust design and operations of hydrocarbon biofuel supply chain integrating with existing petroleum refineries considering unit cost objective. Comput. Chem. Eng..

[56] Salas S.D., Geraili A., Romagnoli J.A. (2017). Optimization of Renewable Energy Businesses under Operational Level Uncertainties through Extensive Sensitivity Analysis and Stochastic Global Optimization. Ind. Eng. Chem. Res..

[57] Ng R.T.L., Maravelias C.T. (2017). Design of biofuel supply chains with variable regional depot and biorefinery locations. Renew. Energy.

[58] Babazadeh R. (2018). Robust Optimization Method to Green Biomass-to-Bioenergy Systems under Deep Uncertainty. Ind. Eng. Chem. Res..

[59] Mousavi Ahranjani P., Ghaderi S.F., Azadeh A., Babazadeh R. (2018). Hybrid Multiobjective Robust Possibilistic Programming Approach to a Sustainable Bioethanol Supply Chain Network Design. Ind. Eng. Chem. Res..

[60] Rabbani M., Saravi N.A., Farrokhi-Asl H., Lim S.F.W.T., Tahaei Z. (2018). Developing a sustainable supply chain optimization model for switchgrass-based bioenergy production: A case study. J. Clean. Prod..

[61] Santibañez-Aguilar J.E., Morales-Rodriguez R., González-Campos J.B., Ponce-Ortega J.M. (2016). Stochastic design of biorefinery supply chains considering economic and environmental objectives. J. Clean. Prod..

[62] Sardashti Birjandi M.R., Shahraki F., Birjandi M.S., Nobandegani M.S. (2014). Application of global optimization strategies to refinery hydrogen network. Int. J. Hydrog. Energy.

[63] Ghaderi H., Pishvaee M.S., Moini A. (2016). Biomass supply chain network design: An optimization-oriented review and analysis. Ind. Crop. Prod..

[64] Kraus R.S. (2011). Petroleum Refining Process, Oil and Natural Gas. Encycl. Occup. Health Safety.

[65] Fogassy G., Thegarid N., Toussaint G., van Veen A.C., Schuurman Y., Mirodatos C. (2010). Biomass derived feedstock co-processing with vacuum gas oil for second-generation fuel production in FCC units. Appl. Catal. B Environ..

[66] An H., Wilhelm W.E., Searcy S.W. (2011). Biofuel and petroleum-based fuel supply chain research: A literature review. Biomass Bioenergy.

[67] Isoni V., Kumbang D., Sharratt P.N., Khoo H.H. (2018). Biomass to levulinic acid: A techno-economic analysis and sustainability of biorefinery processes in Southeast Asia. J. Environ. Manag..

[68] Ribas G.P., Hamacher S., Street A. (2010). Optimization under uncertainty of the integrated oil supply chain using stochastic and robust programming. Int. Trans. Oper. Res..

[69] Al-Qahtani K., Elkamel A. (2010). Robust planning of multisite refinery networks: Optimization under uncertainty. Comput. Chem. Eng..

[70] Zuwei L., Jingdai W., Yongrong Y., Azzaro-Pantel C. (2018). Chapter 12 - Robust Engineering Strategy for Solving Optimization Problems of Refinery Hydrogen System. Hydrogen Supply Chains.

[71] Gutierrez G., Galan A., Sarabia D., de Prada C. (2018). Two-Stage Stochastic Optimization of a hydrogen network. Ifac Pap..

[72] Ahmad M.I., Zhang N., Jobson M. (2010). Modelling and optimisation for design of hydrogen networks for multi-period operation. J. Clean. Prod..

[73] Zhou L., Liao Z., Wang J., Jiang B., Yang Y., Hui D. (2013). Optimal design of sustainable hydrogen networks. Int. J. Hydrog. Energy.

[74] Liao Z.-W., Rong G., Wang J.-D., Yang Y.-R. (2011). Rigorous algorithmic targeting methods for hydrogen networks—Part I: Systems with no hydrogen purification. Chem. Eng. Sci..

[75] Zhou L., Liao Z., Wang J., Jiang B., Yang Y. (2014). MPEC strategies for efficient and stable scheduling of hydrogen pipeline network operation. Appl. Energy.

[76] Chen Y., Eslick J., Grossmann I., Miller D., Eden M.R., Siirola J.D., Towler G.P. (2014). Simultaneous Optimization and Heat Integration Based on Rigorous Process Simulations. Computer Aided Chemical Engineering.

[77] Tovar-Facio J., Lira-Barragán L.F., Nápoles-Rivera F., Bamufleh H.S., Ponce-Ortega J.M., El-Halwagi M.M. (2016). Optimal Synthesis of Refinery Property-Based Water Networks with Electrocoagulation Treatment Systems. Acs Sustain. Chem. Eng..

[78] Wang B., Gebreslassie B.H., You F. (2013). Sustainable design and synthesis of hydrocarbon biorefinery via gasification pathway: Integrated life cycle assessment and technoeconomic analysis with multiobjective superstructure optimization. Comput. Chem. Eng..

[79] Hafyan R., Bhullar L., Putra Z., Bilad M., Wirzal M., Nordin N. (2019). Sustainability assessment of xylitol production from empty fruit bunch. Matec Web Conf..

[80] Hafyan R.H., Prasetyo W.D., Bhullar L., Putra Z.A., Bilad M.R., Wirzal M.D.H., Nordin N.A.H.M. (2019). Multi-objective Optimization of Succinic Acid Production from Empty Fruit Bunch. Asean J. Chem. Eng..

[81] Castillo-Landero A., Ortiz-Espinoza A.P., Jiménez-Gutiérrez A. (2019). A Process Intensification Methodology Including Economic, Sustainability, and Safety Considerations. Ind. Eng. Chem. Res..

[82] El-Halwagi A.M., Rosas C., Ponce-Ortega J.M., Jiménez-Gutiérrez A., Mannan M.S., El-Halwagi M.M. (2013). Multiobjective optimization of biorefineries with economic and safety objectives. Aiche J..

[83] Tsao Y.-C., Thanh V.-V., Lu J.-C., Yu V. (2018). Designing sustainable supply chain networks under uncertain environments: Fuzzy multi-objective programming. J. Clean. Prod..

[84] Cambero C., Sowlati T. (2016). Incorporating social benefits in multi-objective optimization of forest-based bioenergy and biofuel supply chains. Appl. Energy.

[85] Grossmann I.E. (2012). Advances in mathematical programming models for enterprise-wide optimization. Comput. Chem. Eng..

[86] Al-Qahtani K., Elkamel A., Ponnambalam K. (2008). Robust Optimization for Petrochemical Network Design under Uncertainty. Ind. Eng. Chem. Res..

[87] Al-Qahtani K., Elkamel A. (2009). Multisite Refinery and Petrochemical Network Design: Optimal Integration and Coordination. Ind. Eng. Chem. Res..

[88] Al-Sharrah G.K., Alatiqi I., Elkamel A. (2002). Planning an Integrated Petrochemical Business Portfolio for Long-Range Financial Stability. Ind. Eng. Chem. Res..

[89] Al-Sharrah G.K., Alatiqi I., Elkamel A., Alper E. (2001). Planning an Integrated Petrochemical Industry with an Environmental Objective. Ind. Eng. Chem. Res..

